# Real-Time Compact Environment Representation for UAV Navigation

**DOI:** 10.3390/s20174976

**Published:** 2020-09-02

**Authors:** Kaitao Meng, Deshi Li, Xiaofan He, Mingliu Liu, Weitao Song

**Affiliations:** 1 Electronic Information School, Wuhan University, Wuhan 430072, China; meng_kaitao@whu.edu.cn (K.M.); xiaofanhe@whu.edu.cn (X.H.); liumingliu@whu.edu.cn (M.L.); wt.song@whu.edu.cn (W.S.); 2 Collaborative Innovation Center of Geospatial Technology, Wuhan 430079, China

**Keywords:** unmanned aerial vehicle, obstacle sensing, compact environment representation, kernel density estimation

## Abstract

Recently, unmanned aerial vehicles (UAVs) have attracted much attention due to their on-demand deployment, high mobility, and low cost. For UAVs navigating in an unknown environment, efficient environment representation is needed due to the storage limitation of the UAVs. Nonetheless, building an accurate and compact environment representation model is highly non-trivial because of the unknown shape of the obstacles and the time-consuming operations such as finding and eliminating the environmental details. To overcome these challenges, a novel vertical strip extraction algorithm is proposed to analyze the probability density function characteristics of the normalized disparity value and segment the obstacles through an adaptive size sliding window. In addition, a plane adjustment algorithm is proposed to represent the obstacle surfaces as polygonal prism profiles while minimizing the redundant obstacle information. By combining these two proposed algorithms, the depth sensor data can be converted into the multi-layer polygonal prism models in real time. Besides, a drone platform equipped with a depth sensor is developed to build the compact environment representation models in the real world. Experimental results demonstrate that the proposed scheme achieves better performance in terms of precision and storage as compared to the baseline.

## 1. Introduction

Driven by the advantages of on-demand deployment, high mobility, and low cost, unmanned aerial vehicles (UAVs) have become appealing solutions for a wide range of commercial and civilian applications over the past few years, including remote sensing [1], search and rescue [2], and surveillance [3]. For a UAV navigating in an unknown environment, obstacle detection is essential, specifically for autonomous navigation. In particular, the environment representation model needs to be quickly built from the sensor carried by the UAV and stored in the onboard memory. Such information can be utilized for path planning, traversability analysis, and exploration [4,5,6]. As the UAVs only have limited storage [7], efficient environment representation is crucial.

The most commonly used environment representation method is to divide space into cubes with an equal size [8,9,10]. This method can simplify the original point cloud data and quickly analyze the surrounding obstacle areas. However, the resolution of the corresponding grid maps has to be carefully designed, since a small resolution grid consumes a large storage space while a large resolution grid may lead to unfavorable errors near the obstacle surfaces [11]. With this consideration, an accurate and compact environment representation model without relying on the grid map needs be developed, so as to provide a concise spatial relationship [12,13] and improve the efficiency of navigation [14], as well as environmental information sharing among the UAVs [15]. Nonetheless, building an accurate and compact three-dimensional (3D) environment representation model from an onboard sensor is highly non-trivial. Specifically, the main challenges are three-fold. Firstly, it is difficult to construct a unified compact model for the obstacles because of their unknown shapes and the sensing noise. In addition, it is quite time-consuming to find all the arbitrarily shaped gaps on the obstacle surfaces and determine whether these environmental details should be removed to reduce storage consumption. Moreover, due to the requirement of the perception latency to process the captured sensor data and the limited computational capability of the UAVs [16,17], only low-complexity algorithms can be applied.

**Figure 1:**
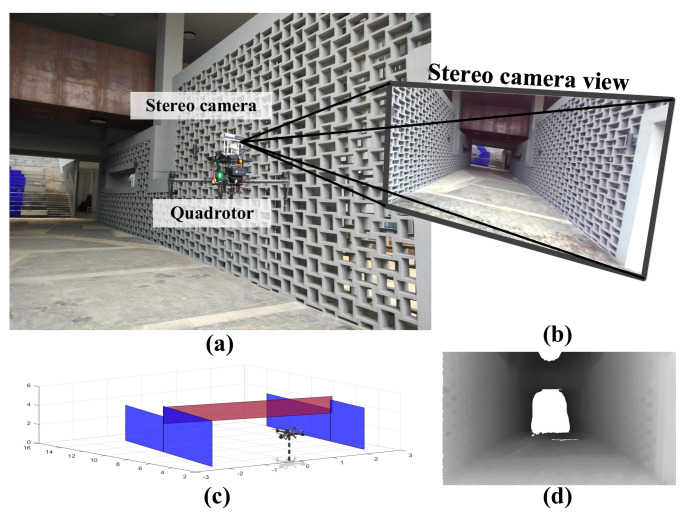
Environment representation experiment. (**a**) Experimental scene. (**b**) Image in the field of view of the UAV. (**c**) Compact model. (**d**) Depth image acquired by the onboard sensor.

To tackle the above challenges, a novel vertical strip extraction algorithm is proposed in this work to analyze the probability density function characteristics of the depth sensor data and extract the obstacle information according to the roughness of the obstacle surface by an adaptive size sliding window. Furthermore, to speed up the elimination of irrelevant environmental details, a two-stage plane adjustment algorithm is presented to quickly fill the irrelevant gaps and then obtain a rectangular outline of the obstacle. Further combining these two algorithms, the obtained overall modeling scheme, dubbed as obstacle surface adaptive plane extraction (OSAPE), can convert the onboard sensor data into the multi-layer polygonal prism models in real time, as shown in Figure 1. In addition, by establishing the compact environment representation model within the field of view, a global map can be obtained by merging the processing results from successive frames and different perspectives.

The main contributions of this work are summarized as follows:

- Two novel algorithms, namely the vertical strip extraction algorithm and the plane adjustment algorithm, are proposed to effectively adapt to different obstacle shapes and different surface roughness, as well as to speed up the elimination of irrelevant environmental details by minimizing redundant information.
- The proposed OSAPE modeling scheme, which is the combination of the two proposed algorithms, can convert the normalized data into simplified prisms based on the size of the UAV in real time.
- By building a drone platform with a depth sensor, real-world experiments are conducted to demonstrate the advantage of the proposed scheme over the baseline.

The rest of this paper is organized as follows. Section 2 discusses the related works. In Section 3, the system model and the main process of the proposed scheme are presented. The vertical strip extraction algorithm and the plane adjustment algorithm are presented in Section 4 and Section 5, respectively. Experimental results are presented in Section 6. In Section 7, conclusions and future works are discussed.

## 2. Related Works

In the literature, several methods about building the compact environment representation models have been developed for unmanned ground robot trajectory generation [18,19,20]. However, two-dimensional (2D) compact environment representation models for the ground robots cannot directly be used in UAV autonomous navigation due to the lack of height information [21]. The extraction of 3D world representations from depth sensors has raised tremendous interests in recent years. For example, in [22], an obstacle detection system in an indoor environment was proposed based on the Kinect sensor, which can accurately detect several types of obstacles in real time. In [23], an omnidirectional obstacle detection method was proposed by repairing the obstacle regions in the depth images, which can provide a three-dimensional omnidirectional obstacle viewing. Most recently, another related research interest in recognizing the obstacles in an unknown environment is semantic SLAM [24], which can provide not only where the obstacles are, but also what they are. Different from these works, the focus of our work is how to further simplify the obstacle model.

Recently, several pioneering works studied the problem of compact environment representation in the context of UAV autonomous navigation. For example, in [25], the polygonal outline of obstacles was extracted based on the grid map, which was used to generate a safe UAV trajectory and avoid the detected obstacles. In [26], an online autonomous collision-free navigation approach was presented by utilizing the octree-based map to generate flight corridors. However, the above grid-based environment representation models result in inflexible grid placement and quantization errors, and the octree-based map [10] requires an additional computational cost to acquire a free space area by searching and querying the state of the grid in tree-based storage structures [27].

There is also some literature on the plane extraction algorithm [28,29,30,31,32]. This algorithm can segment the parts of the depth image or the point cloud data belonging to the same plane, thereby utilizing a small number of geometric parameters to simplify the environment representation. For example, approximate planes are extracted by principal component analysis (PCA) based on voxel maps, and the plane parts are preserved for path planning [28]. However, some irregular obstacle surfaces may be lost during the conversion process due to the preset roughness threshold [29]. Moreover, the farthest recognizable distance of this method is limited (less than 10 m), as the plane fitting parameters cannot be adaptively adjusted according to the distance to the object [30].

Existing works on 3D compact building reconstruction have already demonstrated that the UAV is an effective method to improve the efficiency of reconstruction [33,34]. The observation data are mainly composed of the top area of the objects (e.g., building roofs and the top of the trees). For example, in [35], a modeling method combining meshes and geometric primitives was proposed, which simplifies the environment model while preserving the details of the environments. In [36], a novel algorithm was presented to obtain a lightweight, watertight polygonal surface model from the obtained global point cloud. However, due to different observation angles and global data requirements, these compact building reconstruction methods cannot be directly utilized for real-time obstacle modeling and route planning.

## 3. Adaptive Plane Extraction Model

In this section, the compact environment representation model is presented first, followed by the main process of the proposed modeling scheme.

Consider a UAV equipped with a depth sensor (e.g., binocular vision sensor, ToF camera [37]) performing tasks in an unknown environment. The generated depth data are given by $\tilde{D}\left(u,v\right)$ (The data of other scanning sensors such as LiDAR [38] can also be converted into a 2D matrix $\tilde{D}\left(u,v\right)$), where u=1,...,U and v=1,...V are the column and the row numbers of the sensor data, respectively. $\tilde{D}\left(u,v\right)\in \left[\underline{D},\bar{D}\right]$, where $\underline{D}$ and $\bar{D}$ correspond to the minimum and the maximum measurement distances of the sensor, respectively. For the ease of analysis, the normalized disparity data D(u,v) are defined as:   

(1) $$D\left(u,v\right)=\frac{f^{c}\cdot C}{\tilde{D}\left(u,v\right)},$$

where $f^{c}$ is the camera focal length and *C* is a normalization parameter. Furthermore, the pitch angle θ, the roll angle ϕ, and the yaw angle φ of the camera can be measured by a six degrees of freedom inertial measurement unit (IMU).

A static obstacle in an unknown environment is denoted by $O\in R^{3}$ and can be modeled as a multi-layer polygonal prism $\Theta \in R^{3}$ surrounding this obstacle. The heights of all the layers in Θ are denoted by $Y_{\Theta }=\left(Y_{1},Y_{2},...,Y_{k},...\right)$, where $Y_{k}$ and $Y_{k+1}$ define the height range of the *k*th layer. In addition, the corresponding side profile of the polygonal prism is composed of several vertical rectangles indexed by l∈{1,...,L}, i.e.,

(2) $$\Pi_{l}=\{P_{a}^{1},P_{b}^{2},\eta \}.$$

**Figure 2:**
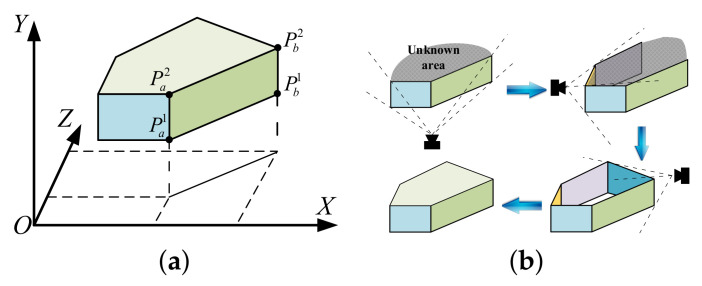
Compact environment representation models. (**a**) Rectangle parameters. (**b**) The modeling process.

In Equation (2), $P_{a}^{1}$ and $P_{b}^{2}$ are two diagonal points of the rectangle $\Pi_{l}$ shown in Figure 2a, and the plane fitting parameter η can provide a basis for merging planes, which will be elaborated later in Section 5.3. In addition, the orientation from the point $P_{a}^{1}$ to the point $P_{b}^{1}$ is utilized to distinguish free space and obstacles without additional parameters. In particular, following the orientation from $P_{a}^{1}$ to $P_{b}^{1}$, the area on the right-hand side is always free space from the top view. On the contrary, the area on the left-hand side is either an obstacle or an unknown area.

**Figure 3:**
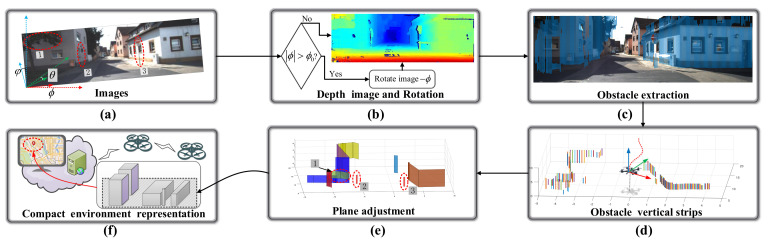
The flowchart of the proposed compact environment representation scheme. (**a**) RGB images. (**b**) Depth image and rotation judgment. (**c**) Obstacle extraction-based probability density function. (**d**) Vertical strips. (**e**) The results of the plane adjustment algorithm. (**f**) The application scenario of the compact environment representation model.

With the above consideration, a novel modeling scheme is proposed to convert the normalized data D(u,v) into several rectangles and merge them into simplified prisms in real time. The main process of the proposed scheme is illustrated in Figure 3. Firstly, the depth image is obtained by a depth sensor show in Figure 3a, and whether the image should be rotated or not is determined by the roll angle ϕ of the camera. Then, the identified 2D obstacles are converted into 3D Euclidean space, shown in Figure 3d, which will be referred to as the vertical strips in the subsequent discussions. The vertical planes are fitted based on the coplanarity of the obtained vertical strips shown in Figure 3e.

Based on the above discussion, the obtained rectangles at multiple viewing angles can be merged and fused to form closed multi-layer polygonal prisms. The modeling process of a single-layer prism is shown in Figure 2b. Specifically, the newly obtained planes are clustered according to the positions of their vertices and merged with adjacent planes. A polygonal prism Θ will be generated if the planes in one cluster form a closed side profile. The proposed modeling scheme mainly consists of the vertical strip extraction algorithm and the plane adjustment algorithm, which will be illustrated in Section 4 and Section 5, respectively, in detail. Important notations and symbols used in this work are given in Table 1.

## 4. The Proposed Vertical Strip Extraction Algorithm

In this section, the vertical strip extraction algorithm is proposed, which identifies the obstacles in the normalized disparity data by columns and converts them into a 3D Euclidean space.

### 4.1. Statistical Estimation of Obstacles

The normalized disparity data D(u,v) of pixels belonging to the same obstacle are approximately equal or within a certain range. Therefore, to improve the anti-noise capability and the accuracy of the obstacle model, the distribution characteristics of the normalized disparity data are analyzed, and the roughness of the obstacle surfaces is estimated statistically.

Kernel density estimation (KDE) [39] is a non-parametric way to estimate the probability density function of random variables, which can be utilized to analyze the distribution characteristics of the obstacles in the sensor data while resisting measurement noise. If the roll angle |ϕ| exceeds the threshold $\phi_{0}$, the disparity data will be rotated by the angle −ϕ to facilitate statistical analysis.

**Lemma** **1.** *Given the normalized disparity data D(u,v) and the pitch angle θ of the UAV, a new converted disparity value that eliminates the influence of the pitch angle is given by:*

(3) $$D_{\theta }\left(u,v\right)=\frac{D\left(u,v\right)\cdot f^{c}}{f^{c}\cdot \cos\theta -\left(v-v_{0}\right)\cdot \sin\theta }.$$

**Proof.**  Please refer to Appendix A. □

After eliminating the influence of the pitch angle of the UAV by Lemma 1, the probability density function of the disparity value *x* in the *u*th column is given by:

(4) $$F_{u}\left(x\right)=\frac{1}{n\cdot h}\sum_{v=1}^{n}K(\frac{x-D_{\theta }\left(u,v\right)}{h}),$$

where K(x) is the kernel function and *h* represents the width of the kernel function. The kernel K(x) satisfies the conditions ∫K(x)dx=1 and K(x)>0. The widely used Gaussian kernel is chosen to estimate the obstacle because of its strong noise immunity,

(5) $$K\left(x\right)=\left\{\begin{array}{c} \frac{A}{\sigma \sqrt{2\pi }}\exp\left(\frac{-x^{2}}{2\sigma^{2}}\right)\\ 0 \end{array}\right.\begin{array}{c} ,\\ , \end{array}\begin{array}{c} \left|x\right|<\frac{1}{2}\\ \mathrm{otherwise} \end{array},$$

where σ is the standard deviation and *A* is a normalization parameter. The center of the Gaussian kernel is $D_{\theta }\left(u,v\right)$, and σ is set according to the sensor noise and depth resolution.

**Figure 4:**
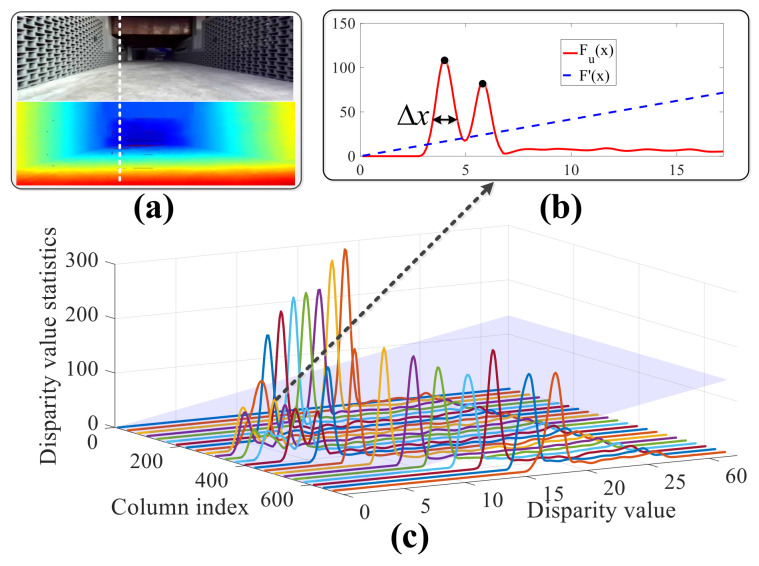
Example of probability density function of the normalized disparity value. (**a**) RGB image and the disparity data. (**b**) Probability density function of one column. (**c**) Probability density function of several columns.

To filter out the measurement noise and slight undulations on the ground, the minimum recognizable obstacle height is set to $H^{m}$, and it is not difficult to verify that the corresponding minimum value of $F_{u}\left(x\right)$ is given by:

(6) $$F^{\prime }\left(x\right)=\frac{H^{m}\cdot x}{C},$$

where *x* is the corresponding disparity value of the obstacle and *C* is the normalization parameter used in Equation (1). As shown in Figure 4, several columns of the probability density function are displayed in different colors, and the blue translucent plane represents the minimum threshold $F^{\prime }\left(x\right)$ under different disparity values. The peak of the probability density function is greater than $F^{\prime }\left(x\right)$, which indicates that there exists an obstacle near the current disparity value *x*. The probability density function in Figure 4b corresponds to the white dotted line in Figure 4a.

### 4.2. Obstacle Identification with a Sliding Window

Due to the measurement error of the depth sensor and the narrow gaps on the obstacle surface, depth pixels may be partially mutated, resulting in the same obstacle being divided into several parts. To address this issue, the sliding windows with adaptive sizes are utilized to identify the obstacles in the normalized disparity data, and the corresponding window size is adjusted according to the roughness of the obstacle surfaces to improve the anti-noise capability.

Specifically, the number and the distance of the obstacles in the *u*th column can be determined by the peak value $F_{u}\left(x_{i}\right)$ above the threshold $F^{\prime }\left(x\right)$, where $x_{i}$ is the disparity value of the *i*th identified obstacle in the column *u*. Construct one sliding window for each disparity value $x_{i}$. The width of the corresponding sliding window is given by $\Delta x_{i}=\left|{\bar{x}}_{i}-{\underline{x}}_{i}\right|$, where ${\bar{x}}_{i}$ and ${\underline{x}}_{i}$ are computed by $F_{u}\left(\tilde{x}\right)=\alpha \cdot F_{u}\left(x_{i}\right)$ for $\tilde{x}={\bar{x}}_{i}$ and $\tilde{x}={\underline{x}}_{i}$, respectively, and $\Delta x_{i}$ reflects the roughness of the obstacle surface. Furthermore, the length $\Delta v_{i}$ of the *i*th sliding window is adjusted according to the minimum height $H^{s}$ of a passable region of the UAV as follows:

(7) $$\Delta v_{i}=\frac{H^{s}\cdot g\left(x_{i}\right)}{C}.$$

In Equation (7), the estimated disparity value $g(x_{i})$ is designed according to the influence of the measurement noise, which is given by:

(8) $$g\left(x\right)=\frac{C\cdot f^{c}}{\frac{C\cdot f^{c}}{x}-k_{e}\cdot {\left(\frac{C\cdot f^{c}}{x}\right)}^{2}},$$

where $k_{e}$ is the proportional coefficient of sensor measurement error. Intuitively, the estimated disparity value $g(x_{i})$ corresponds to the minimum distance to the obstacle under the influence of noise, so that the window height can be adjusted according to the measurement noise. More specifically, the noise increases approximately proportional to the square of the distance according to the measurement characteristics of the depth sensor [40].

To speed up identification, the sliding step is set to half the length of the corresponding window. As the window slides down, the state of the pixel $D_{\theta }\left(u,v\right)$ can be determined by the following conditions:

1. The average of the disparity value in the sliding window is within the range $\left[{\bar{x}}_{i},{\underline{x}}_{i}\right]$ and
2. more than half of the pixels in the sliding window are within the range $\left[{\bar{x}}_{i},{\underline{x}}_{i}\right]$.

If both conditions are admitted, the pixels in the sliding window belong to the *i*th obstacle. Otherwise, the last pixel of the disparity value within the range $\left[{\bar{x}}_{i},{\underline{x}}_{i}\right]$ is set as the endpoint of the obstacle.

**Figure 5:**
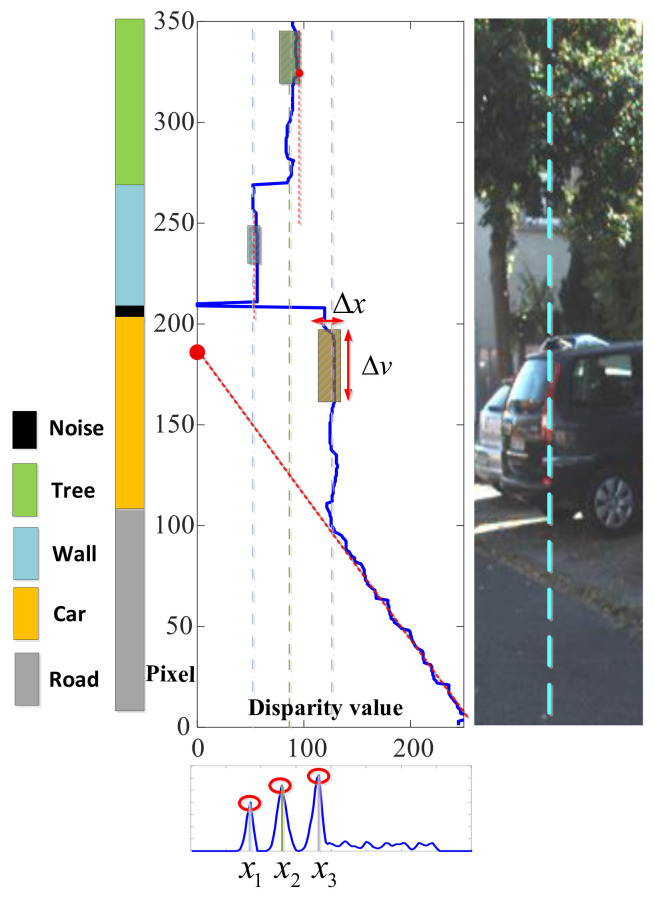
Illustration of obstacle identification with sliding windows.

Based on the above discussion, the obstacle detected in the column *u* is described as $\left({\hat{x}}_{i},u,v_{i}^{b},v_{i}^{t}\right)$, where ${\hat{x}}_{i}$ is the estimated disparity value of the obstacle $x_{i}$; $v_{i}^{t}$ and $v_{i}^{b}$ are the top and the bottom coordinates of obstacles in the disparity data, respectively. In particular, the estimated disparity value ${\hat{x}}_{i}$ is determined by the roughness of the obstacle surfaces, which is given by:

(9) $${\hat{x}}_{i}=\left\{\begin{array}{cc} \max_{v}{\left\{D_{\theta }\left(u,v\right)\right\}}_{v\in \{v_{i}^{b},...,v_{i}^{t}\}}, & \Delta x_{i}>e\left(x_{i}\right)\\ \frac{1}{∥v_{i}^{t}-v_{i}^{b}∥+1}\cdot \sum_{v\in \{v_{i}^{b},...,v_{i}^{t}\}}\{D_{\theta }\left(u,v\right)\}, & \Delta x_{i}\leq e\left(x_{i}\right) \end{array}\right.,$$

where $e\left(x\right)=k_{e}\cdot {\left(\frac{C\cdot f^{c}}{x}\right)}^{2}+2\alpha \cdot h$. For example, Figure 5 demonstrates this sliding process. Specifically, the horizontal and the vertical units in Figure 5 correspond to the normalized disparity value and the row index of the depth data, respectively. Since $\Delta x_{i}$ for the tree is greater than its corresponding threshold $e(x_{i})$, the distance to the object is represented by the closest point. On the contrary, for a relatively smooth wall, its distance is estimated by the average disparity value to improve the estimation accuracy.

With the above process, the obstacle surfaces with relatively concentrated depth values can be identified. However, the obstacles with an irregular structure or scattered depth values may not be extracted by this method. To build a unified obstacle model, a discretization strategy is presented in the next subsection to segment the remaining pixels.

### 4.3. Irregular Object Processing

In this subsection, the horizontal surface in the remaining pixels is identified first, followed by irregular or inclined object segmentation.

**Figure 6:**
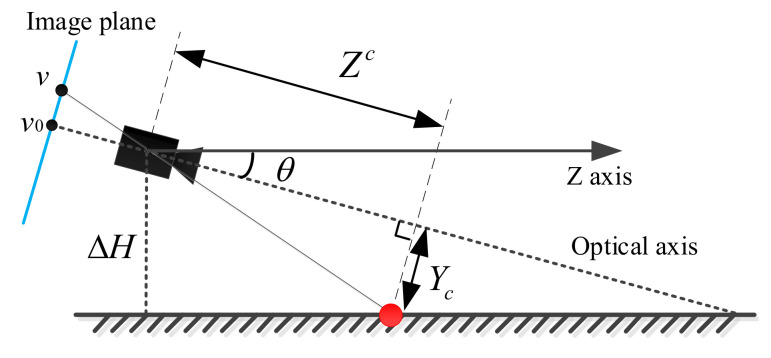
The geometric relationship of the horizontal surface.

The geometric relationship of the horizontal surface in the camera coordinate system is given as follows:

(10) $$Z^{c}=\frac{\Delta H}{\sin\theta }-\frac{Y^{c}}{\tan\theta }=\frac{f^{c}\cdot C}{D(u,v)},$$

and:

(11) $$\frac{v-v_{0}}{f^{c}}=\frac{Y^{c}}{Z^{c}},$$

where $Z^{c}$ and $Y^{c}$ are the horizontal distance and the vertical distance to a point on the horizontal plane (cf. the red circle in Figure 6), respectively. In Equations (10) and (11), ΔH is the altitude difference between the horizontal surface and the depth sensor shown in Figure 6, and the image calibration center is $\left(u_{0},v_{0}\right)$. By plugging Equation (11) into Equation (10), the row index *v* for the horizontal surface is given as follows:

(12) $$v=v_{0}+\frac{\Delta H}{C\cdot \cos\theta }\cdot D\left(u,v\right)-f^{c}\cdot \tan\theta .$$

If D(u,v)=0, then $v=v_{0}-f^{c}\cdot \tan\theta$, which corresponds to the vanishing point. For example, it can be seen from Figure 5 that the extension of the ground portion in the v-disparity image will pass through a fixed point. Hence, a horizontal surface can be identified by fitting the correlation coefficient of the normalized disparity data D(u,v) and the row index *v*. The fitted image center is given by:

(13) $$v^{c}=\frac{\bar{v}\cdot \sum_{v}D{\left(u,v\right)}^{2}-\bar{D}\left(u,v\right)\sum_{v}\left(D(u,v)\cdot v\right)}{{\sum_{v}D(u,v)}^{2}-n_{r}\cdot \bar{D}{\left(u,v\right)}^{2}},$$

and the correlation coefficient between the disparity value and the row index is given by:

(14) $$r=\frac{\sum_{v}\left(D\left(u,v\right)-\bar{D}\left(u,v\right)\right)\cdot \left(v-\bar{v}\right)}{\sqrt{{\sum_{v}\left(D\left(u,v\right)-\bar{D}\left(u,v\right)\right)}^{2}\cdot \sum_{v}{\left(v-\bar{v}\right)}^{2}}},$$

where $n_{r}$ is the number of pixels to be identified and $\bar{v}=\frac{\sum v}{n_{r}}$, $\bar{D}\left(u,v\right)=\frac{\sum D(u,v)}{n_{r}}$. The pixels belong to a horizontal surface if $\left(1-\left|r\right|\right)<\varepsilon_{r}$ and $\left|v^{c}-\left(v_{0}-f^{c}\cdot \tan\theta \right)\right|<\varepsilon_{v}$. The horizontal object surface is used as a reference for the thickness of the obstacle in this work.

Then, the pixels that do not meet the above conditions should belong to a sloping or irregular surface. However, it is difficult to obtain the accurate position of these obstacles and build a unified compact model. Here, a simple and effective strategy is presented to quickly divide the remaining pixels at a constant height interval $H^{d}$, which is set according to the resolution requirements. Similar to Equation (6), the number of segmented pixels can be estimated as $n^{d}=H^{d}\cdot x/C$, where *x* is the disparity value of the irregular object. The segmentation position of the irregular obstacles can be obtained by searching in steps of $n^{d}$. As a result, the pixels belonging to the irregular objects in the same column are divided into multiple segments along the vertical direction, and the closest point in each segment is used to represent the distance to the divided object. This simple and effective way can convert different types of objects into arrays with four elements, i.e., $\left({\hat{x}}_{i},u,v_{i}^{b},v_{i}^{t}\right)$.

### 4.4. Vertical Strip Clustering

In this subsection, the identified obstacle in the normalized disparity data will be converted into 3D Euclidean space. In particular, the projection of a point $P^{c}=\left(X^{c},Y^{c},Z^{c}\right)$ from the camera coordinate system to the image coordinate system is given by:

(15) $$\left[\begin{array}{c} u\\ v\\ 1 \end{array}\right]=\left[\begin{array}{ccc} \frac{D(u,v)}{C} & 0 & \frac{D\left(u,v\right)\cdot u_{0}}{C\cdot f^{c}}\\ 0 & \frac{D(u,v)}{C} & \frac{D\left(u,v\right)\cdot v_{0}}{C\cdot f^{c}}\\ 0 & 0 & \frac{D(u,v)}{C\cdot f^{c}} \end{array}\right]\left[\begin{array}{c} X^{c}\\ Y^{c}\\ Z^{c} \end{array}\right].$$

Furthermore, the point $P^{c}$ in the camera coordinate system is given by:

(16) $${\left[X^{c},Y^{c},Z^{c},1\right]}^{T}=\left[\begin{array}{cc} R & T\\ 0 & 1 \end{array}\right]\cdot {\left[X^{w},Y^{w},Z^{w},1\right]}^{T},$$

where *R* and *T* denote the rotation matrix and the translation matrix of the camera. According to Equations (15) and (16), $\left(\hat{x},u,v_{i}^{b},v_{i}^{t}\right)$ in the camera coordinate system is converted into 3D Euclidean space, i.e., $S_{i}=\left(X_{i},Y_{i}^{b},Y_{i}^{t},Z_{i}\right)$, which looks like a strip perpendicular to the ground. To further build the compact contours of the obstacle, the spatially adjacent vertical strips should be clustered.

The clustering of the vertical strips in 3D Euclidean space often requires a radius search to find their neighbors within the radius. However, the 3D radius search is time consuming when the amount of data is large. By utilizing the spatial continuity of adjacent pixels on the normalized disparity data, the vertical strips are clustered according to the 2D index on these disparity data, which can improve operational efficiency. Specifically, the distance between the vertical strip and the last inserted strip in each cluster is calculated. If the distance is less than the minimum width $W^{s}$ of the passable region, this vertical strip will be inserted into the corresponding cluster. Otherwise, a new cluster is created for this vertical strip. The details of the fast strip clustering algorithm are given in Algorithm 1.
**Algorithm 1** Fast strip clustering algorithm.
1:Initialize cluster set $C_{1}=\left\{S_{1}\right\}$, R=1.2:**for**u=1 to *U* **do**3: **for** each $S_{i}$ in column *u* **do**4:  Calculate the distance $d_{i,C_{r}}$ from this strip to the last inserted strip in each cluster $C_{r}$, wherer∈{1,...,R}.5:  **if** $\min_{r}d_{i,C_{r}}<d^{th}$ **then**
6:   Insert strip $S_{i}$ into cluster $C_{r}$.7:  **else**
8:   R=R+1, create a new cluster $C_{R}$.9:   Insert strip $S_{i}$ into the new cluster $C_{R}$.10:  **end if**
11: **end for**
12:**end for**



### 4.5. Computational Complexity

For a depth sensor data with size n=U·V, the calculation time of the probability density function $t_{1}\propto \left\lceil n\cdot h\right\rceil$ with the ceiling operator $\left\lceil \cdot \right\rceil$ and the kernel width *h*. When the height of the sliding window is *M*, the calculation time of the sliding extraction $t_{2}\propto \left\lceil 2\cdot n/M\right\rceil$. The time of the remaining pixels processing $t_{3}\propto n^{r}\left(n^{r}\leq n\right)$, where $n^{r}$ is the number of remaining pixels. In addition, the computational complexity for the projection of the vertical strips is negligible, since the number $n^{s}$ of strips admits $n^{s}\ll n$. Therefore, the complexity of the vertical strip extraction algorithm is O(n).

## 5. The Proposed Plane Adjustment Algorithm

In this section, the plane adjustment algorithm is proposed to convert the obtained vertical strips into prisms, while useless environmental details will be removed. Since the gap shape on the obstacle surface is arbitrary and unknown, it will take a long time to search every gap and determine whether the UAV can pass. To avoid this, a two-stage adjustment method is presented: first, fill the gap in the vertical direction, and then, convert the concave surface in the horizontal direction. These two main procedures are elaborated in Section 5.1 and Section 5.2, respectively.

### 5.1. Vertical Gap Filling

**Figure 7:**
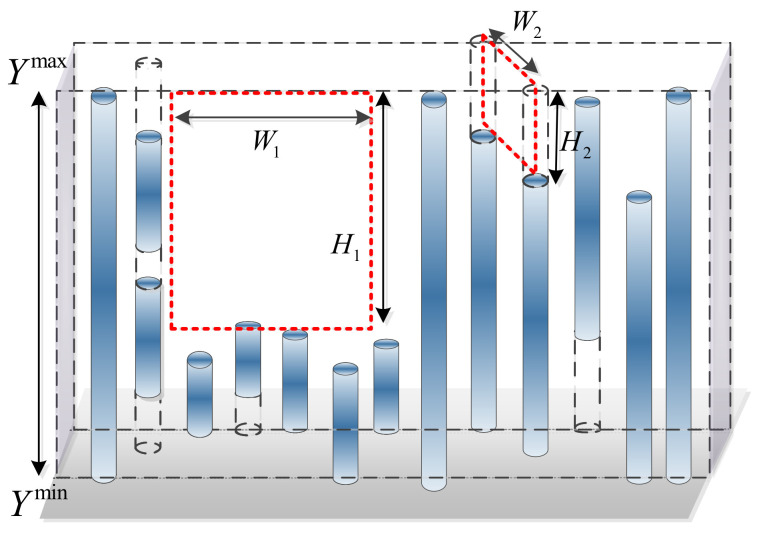
Illustration of the vertical gap filling method.

The vertical strips belonging to the same obstacle can be regarded as a wall with a certain thickness as shown in Figure 7, in which the translucent blue cylinders represent the vertical strips. The height of this wall is determined by the maximum value $Y^{\max}$ and the minimum value $Y^{\min}$ of the vertical strips in the vertical direction. Obviously, if a narrow gap on the wall is smaller than the minimum size of the passable region, this gap should be filled, as these details are unnecessary for UAV navigation. As a result, the main work of removing unnecessary gaps is to obtain the maximum gap area among the vertical strips.

It is not difficult to find that in the vertical direction, the height of the gaps is the relative complement of each vertical strip $S_{i}=\left(X_{i},Y_{i}^{b},Y_{i}^{t},Z_{i}\right)$ in ${\tilde{S}}_{i}=\left(X_{i},Y_{i}^{\min},Y_{i}^{\max},Z_{i}\right)$. Specifically, there  exists two relative complements, i.e., $S_{i}^{1}=\left(X_{i},Y_{i}^{\min},Y_{i}^{b},Z_{i}\right)$ and $S_{i}^{2}=\left(X_{i},Y_{i}^{t},Y^{\max},Z_{i}\right)$ (cf. the cylinder with the dotted line in Figure 7). If the ranges of these two relative complements are less than $H^{s}$, the relative complements should be filled. Otherwise, the size of the gap can be obtained by calculating the intersection range of the adjacent complements in the vertical direction. Then, a gap smaller than the minimum size of the passable region will be filled, while reserved gaps split the wall into multiple clusters (If the width of the wall is less than the minimum width $W^{s}$ of the passable region, there is no need to search inside this cluster. Therefore, a binary searching strategy is adopted to calculate the complements of the vertical strips, which can greatly reduce the amount of calculation). Hence, the vertical strips inside one cluster are filled with the same height.

### 5.2. Concave Surface Converting

Based on the vertical strips obtained in the previous subsection, a concave surface converting algorithm is presented to remove the concave surface according to the size of the passable region of the UAV and obtain the compact obstacle model.

Since the vertical strips in a cluster share the same height after vertical filling adjustment, plane extraction is equivalent to a line segment extraction process with a certain height. However, vertical strips belonging to an uneven surface may be split into more scattered groups, which makes the obstacle model less compact. Inspired by the split and merge algorithm developed in [41] (The core idea of the split and merge algorithm is to iteratively split the entire cluster at the point with the maximum fitting error and merge adjacent parallel line segments), a concave surface converting algorithm is proposed to model uneven surfaces and reduce irrelevant detailed information. Whether it is necessary to delete redundant nodes can be determined by the vector cross product and the distance among three adjacent points. Specifically, for three adjacent vertical strips $S_{i-1}$, $S_{i}$, and $S_{i+1}$, if $\vec{S_{i-1}S_{i}}\times \vec{S_{i}S_{i+1}}>0$ and $\sqrt{{\left(X_{i-1}-X_{i+1}\right)}^{2}+{\left(Z_{i-1}-Z_{i+1}\right)}^{2}}<W^{s}$, the strip $S_{i}$ will be deleted; here, $\vec{S_{i-1}S_{i}}$ represents the horizontal vector from $S_{i-1}$ to $S_{i}$, and $\vec{S_{i-1}S_{i}}\times \vec{S_{i}S_{i+1}}$ denotes a cross product operation to determine the concavity. The above steps are repeatedly performed until no improvement can be made. The detailed steps of concave surface converting algorithm are illustrated in Algorithm 2.

### 5.3. Adjacent Plane Refinement

Due to different observation angles and fitting errors of the extracted obstacle contours, the adjacent planes belonging to the same object may overlap or intersect. With this consideration, a plane refinement strategy is presented to extend the adjacent planes and remove redundant parts and then obtain an interconnected prism profile. Specifically, the adjacent planes are supposed to be merged if the following two conditions are satisfied:

1. The angle between the two planes is less than the threshold $\varphi_{0}$ and
2. the distance between the boundaries of the adjacent sides is smaller than $W^{s}$.

**Algorithm 2** Concave surface converting algorithm.
1:Initialize line set L=∅, and put all strips into set $\Omega_{1}$ and R=1.2:**for** each $\Omega_{r}$ **do**3: **if** $|\Omega_{r}|>3$ **then**
4:  The strips in $\Omega_{r}$ are fitted into a line according to their horizontal locations with the fitted error $\varepsilon^{l}$.5:  **if** $\varepsilon^{l}<=\varepsilon^{th}$ **then**
6:   Put this line into *L*, and remove the corresponding strips in $\Omega_{r}$.7:  **else**
8:   Split the strips at the maximum fitting error into $\Omega_{R+1}$ and $\Omega_{R+2}$; R=R+2.9:  **end if**
10: **else if**$\vec{S_{i-1}S_{i}}\cdot \vec{S_{i+1}S_{i}}>0$ and $\sqrt{{\left(X_{i-1}-X_{i+1}\right)}^{2}+{\left(Z_{i-1}-Z_{i+1}\right)}^{2}}<W^{s}$ **then**11:  Delete $S_{i}$; put the line composed of $S_{i+1}$ and $S_{i-1}$ into *L*.12:**end if**
13: **end for**
14:Merge collinear line segments in *L*.


To improve the efficiency of the plane fitting, the parameters of a new plane can be calculated based on the original parameters as follows:

(17) $$\eta_{new}=\frac{n_{1}^{s}}{n_{1}^{s}+n_{2}^{s}}\eta_{1}+\frac{n_{2}^{s}}{n_{1}^{s}+n_{2}^{s}}\eta_{2},$$

where $\eta =(n^{s},\bar{X},\bar{Z},\bar{XZ},\bar{XX})$; here, $n^{s}$ represents the number of the strips that are fitted into the plane, and $\bar{X}$, $\bar{Z}$, $\bar{XZ}$, and $\bar{XX}$ denote the corresponding average coordinate values of the vertical strips. Hence, the newly acquired plane can be quickly merged with the established compact model without storing all the merged planes.

### 5.4. Computational Complexity

In the plane extraction process, the computational complexity of the vertical gap filling step is $O(\log n^{s})$, and the complexity of the concave surface converting step is $O({\left(n^{s}\right)}^{2})$. As $O(\log n^{s})$ is negligible as compared to $O({\left(n^{s}\right)}^{2})$, the complexity of the overall proposed scheme is $O\left(n\right)+O({\left(n^{s}\right)}^{2})$.

## 6. Experiment and Analysis

**Figure 8:**
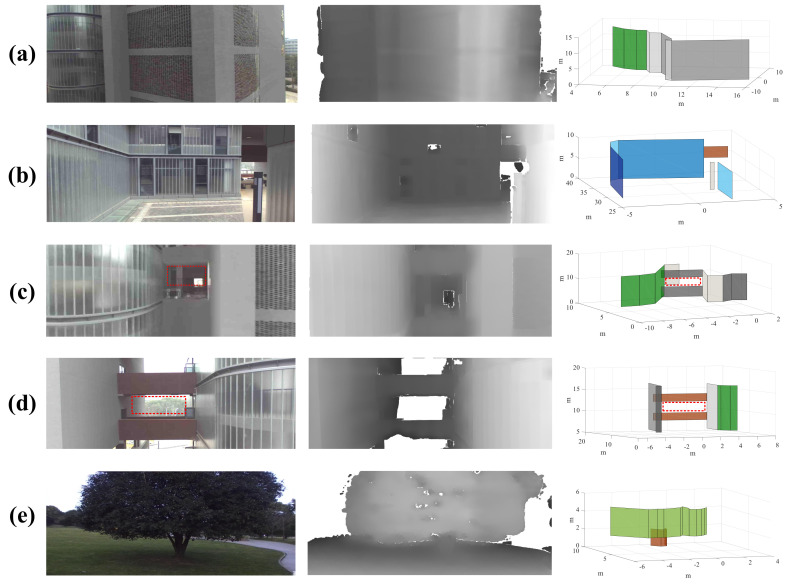
Compact environment representation obtained by the developed drone platform. (**a**) Building outline; (**b**) Alley; (**c**) Balcony; (**d**) Corridor; (**e**) Tree.

In this section, numerical results are provided to evaluate the performance of the proposed algorithms. The simulation results on the AirSim simulator (The AirSim simulator is a photorealistic game engine with cutting-edge graphics features such as high-resolution textures and realistic lighting and shadows, where the depth image can be obtained in real time) and the experiment results on the developed drone platform are presented in Section 6.1 and Section 6.2, respectively. Specifically, the proposed scheme in this work was implemented in C++11, and the simulations were performed on an Intel Core i7-8700K processor. Furthermore, the drone platform was built based on DJI Matrice 100, which is equipped with a ZED binocular vision sensor and can build the compact environment representation model of the real world as shown in Figure 1 and Figure 8. The image size obtained from the AirSim simulation platform [42] is 640 × 480, and the image size of the ZED sensor is 672 × 376.

In the simulation and experiment, the minimum height $H^{s}=1$ m and the minimum width $W^{s}=2$ m of the passable region of the UAV are set according to the size of the drone platform (The height of the drone platform is 0.7 m, and the width of the drone platform is 1.4 m. $H^{s}$ and $W^{s}$ can be set according to the size of the drone platform and the accuracy of the flight controller). The height division range was set to 2 m according to the resolution requirements [25]. Furthermore, the coefficient of the measurement error $k_{e}=0.01$, and the threshold of the fitting error $\varepsilon_{l}=0.2$ m (Based on our experience in the experiments, when $\varepsilon_{l}$ changes within [0.1 m, 0.3 m], there is little difference in the obtained models). In addition, the threshold of the roll angle $\phi_{0}=2^{\circ }$. The proposed OSAPE scheme is compared with the 3D prisms (3DP) scheme [25] in terms of storage, precision, and processing time.

### 6.1. AirSim Simulation

In this subsection, the compact environment representation model on the AirSim simulator is presented first (A video showing the simulations can be found at https://youtu.be/TketUNf0ers), followed by the comparison of storage, precision, and processing time.

#### 6.1.1. Compact Model

**Figure 9:**
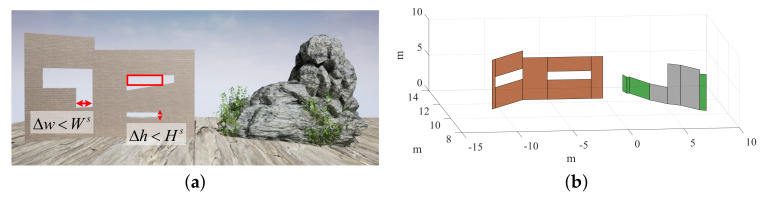
Compact environment representation in the AirSim simulator. (**a**) Obstacle scene. (**b**) Compact environment representation.

**Figure 10:**
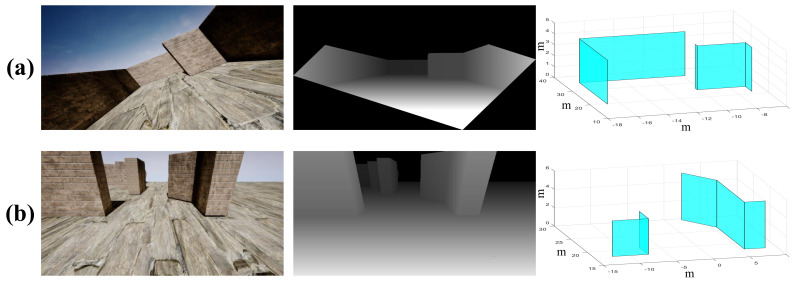
Compact environment representation at large roll and pitch angles. For (**a**), $\phi =30^{\circ }$ and $\theta =0^{\circ }$, and for (**b**), $\phi =0^{\circ }$ and $\theta =30^{\circ }$.

The proposed scheme is evaluated on different kinds of obstacles in this subsection, including buildings, trees, and other man-made objects, some of which are shown in Figure 9 and Figure 10 (The colors of the planes are artificially labeled for the sake of clarity), where the coordinate unit of the 3D compact environment model is meters. For example, it can be seen from Figure 9 that irregular objects (e.g., stones) are modeled as a plurality of planes surrounding them, and gaps in the field of view that are smaller than the passable region of the drone are filled. In Figure 10, the left, the middle, and the right sides are the RGB images, the disparity data, and the corresponding compact environment representation models, respectively. Specifically, Figure 10 shows the obtained models when the drone is at different pitch and roll angles, which indicates that the proposed scheme is adaptable to large tilt angles.

#### 6.1.2. Memory Usage and Model Precision

The proposed scheme is compared with the 3DP scheme [25] for storage consumption and model precision in this subsection (In 3DP, the grid map is obtained from the depth data, and then the contour of the compact model is extracted based on this grid map). Different grid resolutions of the 3DP scheme were set for a clearer comparison, i.e., 0.1 m, 0.2 m, 0.4 m, and 0.8 m.

**Figure 11:**
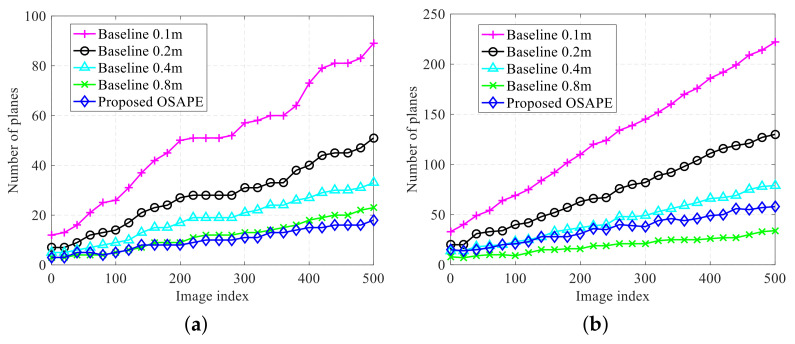
Storage consumption comparison of different schemes in the AirSim simulator. (**a**) Storage of structured obstacle. (**b**) Storage of unstructured obstacles.

First, the performance of storage consumption was evaluated on the AirSim simulator, and the compact obstacle model was obtained in real time. It can be seen from Figure 11 that, as the grid size increases, the storage consumption of the 3DP scheme decreases, which conforms to the discussions in the Introduction. In the AirSim simulator, a compact environment representation model is constructed by gradually merging and fusing the obtained models of multiple frames. The storage consumption for structured obstacles and unstructured obstacles is compared in Figure 11a,b, respectively (The structured obstacles refer to the obstacles with planar surfaces while the unstructured obstacles refer to the obstacles with rough surfaces). For structured obstacles, the proposed OSAPE scheme extracts the planes without quantization, and the unrelated obstacle details are removed; thus, the number of planes is smaller as compared to that obtained by the 3DP scheme. For unstructured obstacles, the number of planes is slightly larger than the 3DP scheme with 0.8 m grids and far smaller than the 3DP scheme with 0.1 m grids.

In addition, the modeling accuracy is defined as the difference between the established compact model and the actual obstacle surfaces. However, the location of obstacles is unknown, and it is difficult to accurately measure the position and the shape of the obstacle surface. Therefore, for quantitative analysis, the modeling accuracy can be approximated by the average distance from the points of the obstacle surfaces to the obtained compact model.

**Figure 12:**
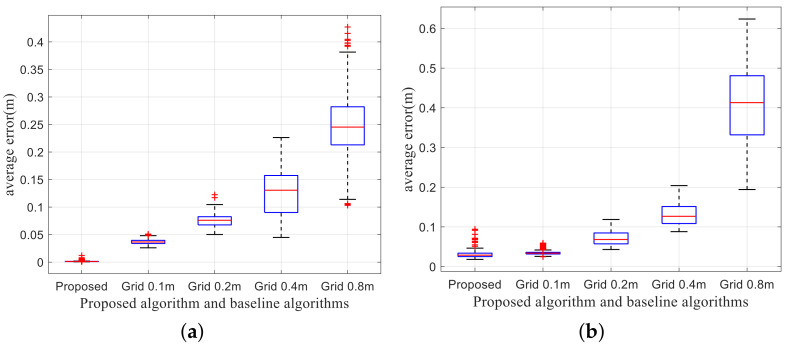
Modeling precision comparison in the AirSim simulator. (**a**) Modeling precision of structured obstacles. (**b**) Modeling precision of unstructured obstacles.

Based on the above discussion, the statistics of the modeling accuracy of the proposed scheme and the 3DP scheme are compared in Figure 12. For the 3DP scheme, as the grid size increases, the modeling accuracy of the 3DP scheme also increases due to the quantization error. For structured obstacles, the modeling error of the proposed scheme is much lower than that of the 3DP scheme with 0.1 m grids. The main reason is that the grid in the 3DP scheme cannot be placed arbitrarily, and the extracted polygon depends on the border of the grids. However, for unstructured obstacles, the modeling precision of the proposed scheme is slightly larger than the 3DP scheme with 0.1 m grids, which is because the proposed scheme eliminates irrelevant obstacle details and introduces modeling error.

#### 6.1.3. Processing Time

**Figure 13:**
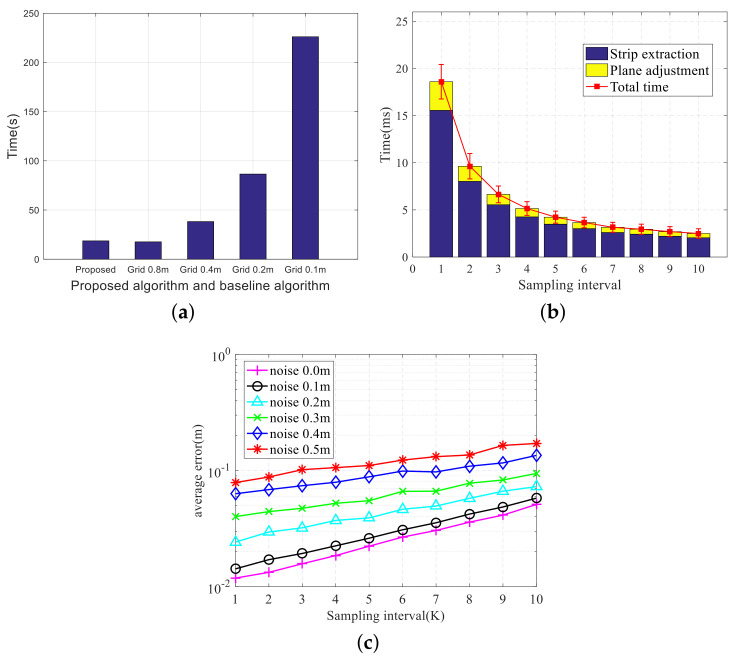
Comparison of processing time and modeling error with different sampling intervals. (**a**) Processing time of different schemes. (**b**) Processing time of different sampling intervals. (**c**) Modeling error of different sampling intervals.

In this subsection, the processing time of the proposed scheme is compared with the 3DP scheme in the AirSim simulator. The processing times of the 3DP scheme with 0.1 m grids and 0.8 m grids are on average about 226.1 ms and 17.6 ms, respectively, as shown in Figure 13a (Since there is no provided source code, the 3DP algorithm is implemented according to [25]). In addition, it can be seen from Figure 13a that, as the grid size increases, the processing time of one frame gradually increases. As shown in Figure 13b, the proposed scheme takes only 18.6 ± 3.0 ms to process one frame of depth sensor data without downsampling in the simulations. The processing time of the proposed scheme can be reduced by downsampling the depth image in the column direction. As the sampling interval increases, the processing time gradually decreases. Here, the sampling interval refers to the number of interval columns for downsampling the depth data only in the column direction, which provides a way to improve the efficiency of the proposed scheme.

Furthermore, the anti-noise performance of the proposed scheme is evaluated by adding random noise of different amplitudes. Figure 13 illustrates that as the sampling interval increases, the efficiency of the proposed scheme improves significantly, but the modeling accuracy and anti-noise performance decrease. Therefore, the sampling rate can be set according to the requirements of efficiency and precision.

### 6.2. Experiment on the Developed Platform

In this subsection, the proposed OSAPE scheme is evaluated on the developed drone platform. Specifically, the developed drone platform flies at an altitude from a few meters to several tens of meters and converts the depth sensor data into the simplified obstacle models in real time. The modeling results are shown in Figure 8, where the left, the middle, and the right sides are the RGB images, the disparity data, and the corresponding compact environment representation models, respectively. It can be seen from Figure 8 that different obstacles including buildings, street lights, and trees are converted into compact planar models. The red dotted rectangles in Figure 8c,d represent the gaps that the drone can pass through, and their sizes are 2.7 m × 2.0 m and 5.7 m × 2 m, respectively, while the actual sizes of these rectangles are 2.9 m × 2.1 m and 5.8 m × 2.2 m, respectively. The extracted window is slightly smaller than the actual one due to the conservative extraction strategy.

**Figure 14:**
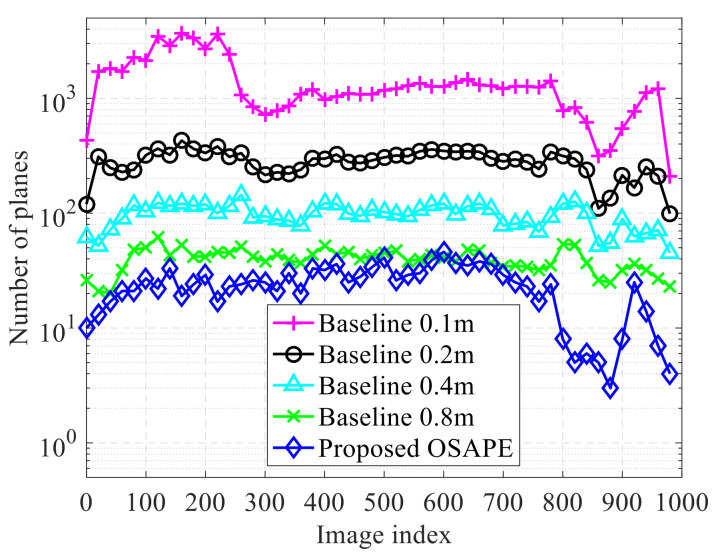
Storage from the developed drone platform. OSAPE, obstacle surface adaptive plane extraction.

The processing time of the proposed scheme is on average about 89ms on the developed drone platform, and the compact environment model can be obtained on the developed drone platform between 10 and 12Hz. Since the processing time of the algorithm has been compared via simulations (cf. Section 6.1.3), the processing time comparison on the developed platform is omitted. In addition, the performance of storage consumption is compared based on the modeling results derived from real flight data collected by the drone platform. Specifically, the proposed scheme reduces the plane numbers of the field of view by up to 40% as compared to the 3DP scheme with 0.8m grids shown in Figure 14.

### 6.3. Application

**Figure 15:**
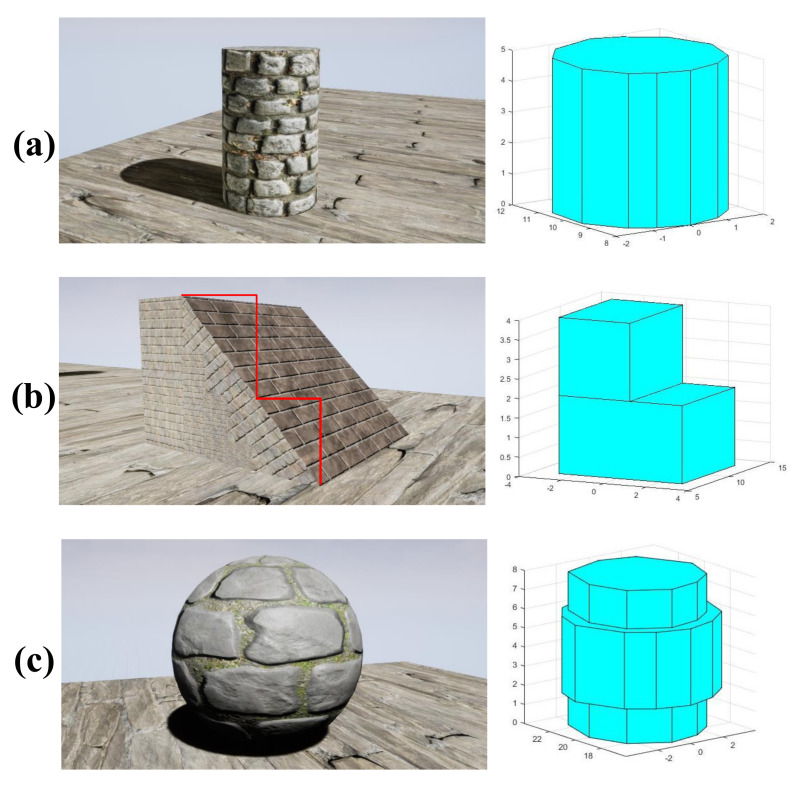
Compact models of obstacles with different shapes. (**a**) Cylinder and its modeling results. (**b**) Trapezoidal prism and its modeling results. (**c**) Sphere and its modeling results.

**Figure 16:**
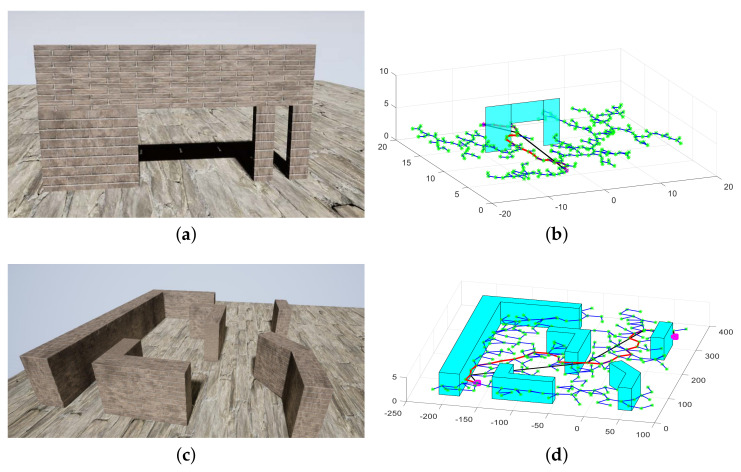
Path planning in the compact environment representation model. RRT, rapidly exploring random trees. (**a**) Obstacle scene. (**b**) RRT in a single frame. (**c**) Obstacle scene. (**d**) RRT in a global map.

In this subsection, the obstacles of different shapes shown in Figure 15 are converted into multi-layer polygonal prisms, including cylinders, trapezoidal prisms, and spheres. Furthermore, the obtained compact environment representation model can be used for some traditional path planning algorithms, such as rapidly exploring random trees (RRT) [43] and probabilistic road map (PRM) [44]. Here, the commonly used RRT method is used to verify the validity of the obtained compact model. RRT is an algorithm designed to search for non-convex space and generate feasible paths by randomly building a space-filled tree. Based on the position of the obtained compact model, it can be determined whether the sampled path point is feasible. As shown in Figure 16, the pink dots represent the starting location and the target location, and the green dots represent the sampling path points on the space-filled tree. In addition, the red and the black lines correspond to the planned path and the smoothed path, respectively. It is worth noticing that the gaps that the UAV cannot pass through are eliminated, so as to avoid some unsafe path generated by the path planning algorithm.

## 7. Conclusions and Future Works

In this paper, the problem of the compact environment representation model for UAV navigation is studied, where the depth data from the onboard sensor are analyzed and converted into the multi-layer polygonal prisms. By analyzing the probability density function of the normalized disparity data, a novel vertical strip extraction algorithm is proposed to improve adaptability to the roughness of the obstacle surfaces and obtain the more accurate locations of the obstacles. Furthermore, the plane adjustment algorithm is presented to speed up the elimination of irrelevant environmental details by minimizing redundant information and obtaining a rectangular outline of the obstacle. By combining these two proposed algorithms, the obtained modeling scheme can convert the depth sensor data into simplified prisms in real time. In addition, a drone platform is developed to build a compact environment representation model in the real world. The experimental results demonstrate that the proposed scheme consumes less storage as compared to the baseline algorithm and provides higher accuracy in modeling structured obstacles.

Extending the proposed scheme to the complex terrains and dynamic scenes, exploring a collaborative multi-UAV modeling strategy, and investigating more efficient storage and search mechanisms in the large mission area are all worthwhile future works. 

## Figures and Tables

**Table 1:** List of notations.

Notation	PhysicalMeaning
$\Pi_{l}$	Vertical rectangle
η	The parameter of the rectangle
θ, ϕ, and φ	The pitch, roll, and yaw angles
D(u,v)	The normalized disparity data
$f^{c}$	Focal length
K(x)	The Gaussian kernel function
$F_{u}\left(x\right)$	The probability density function in column *u*
$F^{\prime }\left(x\right)$	The minimum threshold of the probability density function
$x_{i}$	The center of the *i*th peak
$\Delta x_{i}$	The width of the *i*th sliding window
$\Delta v_{i}$	The height of the *i*th sliding window
$H^{m}$	The minimum recognizable obstacle height
$H^{s}$	The minimum height to the passable region for the UAV
$W^{s}$	The minimum width to the passable region for the UAV
g(x)	Estimated disparity value
$C_{r}$	The *r*th cluster of the vertical strips

**Figure A1:**
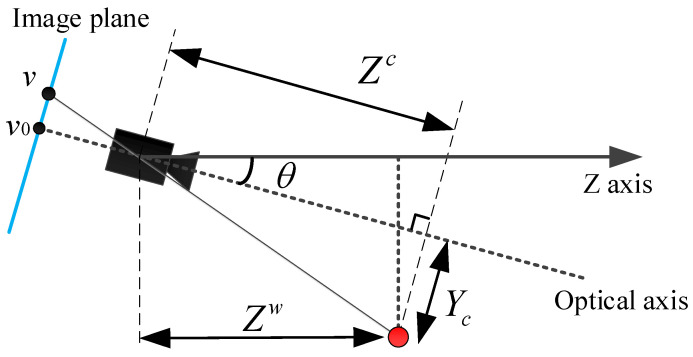
Illustration of the proof of Lemma 1.

## Appendix A. Prove of Lemma 1

The relationship between the coordinate value of the Z-axis in the world coordinate system and that of the camera coordinate system is shown in Figure A1. Under the normalized disparity value D(u,v) and the pitch angle θ of the UAV, it is not difficult to find that the coordinate value $Z^{w}$ in the world coordinate system can be given as:

(A1) $$Z^{w}=Z^{c}\cdot \cos\theta -Y^{c}\cdot \sin\theta .$$

Furthermore, the relationship between *v* and $Y^{c}$ is given as follows:

(A2) $$\frac{v-v_{0}}{f^{c}}=\frac{Y^{c}}{Z^{c}}.$$

By plugging Equations (1) and (A2) into Equation (A1), the disparity value that eliminates the influence of the pitch angle of the UAV follows:

(A3) $$D_{\theta }\left(u,v\right)=\frac{D\left(u,v\right)\cdot f^{c}}{f^{c}\cdot \cos\theta -\left(v-v_{0}\right)\cdot \sin\theta }.$$
